# Pain Assessment and Management in Oncological Practice: A Survey from the Italian Network of Supportive Care in Oncology

**DOI:** 10.3390/healthcare13030212

**Published:** 2025-01-21

**Authors:** Andrea Antonuzzo, Silvia Gonella, Livio Blasi, Simona Carnio, Ciro Franzese, Luigi Marano, Daniele Santini, Paolo Bossi

**Affiliations:** 1 Department of Public Health and Pediatrics, University of Turin, 10126 Turin, Italy; andrea.antonuzzo@istitutotumori.mi.it (A.A.); silvia.gonella@unito.it (S.G.); 2 A.R.N.A.S. Ospedali Civico di Cristina Benfratelli (PA), 90127 Palermo, Italy; livio.blasi@arnascivico.it; 3 Department of Oncology, AOU San Luigi-Orbassano, University of Turin, 10043 Turin, Italy; simona.carnio@libero.it; 4 Department of Radiotherapy and Radiosurgery, IRCCS Humanitas Research Hospital, Via Manzoni 56, 20089 Rozzano, Italy; ciro.franzese@hunimed.eu; 5 Department of Biomedical Sciences, Humanitas University, Via Rita Levi Montalcini 4, Pieve Emanuele, 20072 Milan, Italy; 6 Department of Medicine, Academy of Applied Medical and Social Sciences-AMiSNS: Akademia Medycznych I Spolecznych Nauk Stosowanych, 82-300 Elbląg, Poland; l.marano@amisns.edu.pl; 7 Department of General Surgery and Surgical Oncology, “Saint Wojciech” Hospital, “Nicolaus Copernicus” Health Center, 80-462 Gdańsk, Poland; 8 UOC Oncologia A, Policlinico Umberto I, Università la Sapienza, 00161 Roma, Italy; daniele.santini@uniroma1.it; 9 Department of Medical Oncology and Hematology, IRCCS Humanitas Research Hospital, Via Manzoni 56, 20089 Rozzano, Italy

**Keywords:** cancer pain, opioids, pain management, pain assessment, clinical practice, palliative care, supportive care

## Abstract

**Background/Objectives:** Cancer pain is prevalent across all stages of the disease, significantly impacting patients’ lives. Despite the availability of guidelines, its assessment and management remain suboptimal in many clinical settings. This study aimed to explore how healthcare professionals in Italy assess and manage cancer pain, identifying gaps and educational needs to improve adherence to best practices. **Methods:** A multidisciplinary Scientific Board designed an online survey comprising 28 items addressing demographics, pain assessment tools, perception of pain, pharmacological management, adverse effects, and barriers to care. The survey targeted oncologists, nurses, radiotherapists, and surgeons within the Italian Network of Supportive Care in Oncology. Data were collected from March to May 2024 and analyzed descriptively. **Results:** Eighty-five professionals participated, predominantly oncologists (63.5%). Most respondents utilized pain scales, with the Numerical Rating Scale (60.3%) being the most frequent. However, specific tools like the Edmonton Symptom Assessment System (ESAS) were underutilized, possibly due to limited training and time constraints. Factors influencing analgesic choice included patient comorbidities (30.3%) and polypharmacy (28.0%). The main barriers to effective pain management included inadequate training (85.5%) and poor communication between patients and caregivers (40.6%) and within care teams (31.9%). Preventive measures for opioid-induced adverse events were widely employed, with laxatives (52.7%) and antiemetics (40.5%) being the most common. **Conclusions:** Findings underscore the need for structured training programs, improved communication, and integration of validated assessment tools. A multidisciplinary, proactive approach to cancer pain assessment and management is essential to optimize care and reduce its burden across all disease stages.

## 1. Introduction

Patients with cancer commonly experience pain across all disease stages, resulting in poorer physical and emotional well-being. However, the prevalence of pain increases from >50% during cancer treatment to >65% at advanced, metastatic, or terminal disease [1,2]. The increase in survival rates due to early diagnosis and improved cancer treatment of recent years has resulted in greater numbers of patients experiencing persistent pain due to cancer, related treatments, or both; 5–10% of cancer survivors experience chronic severe pain that interferes significantly with functioning [3,4]. The psychological suffering caused by pain can also worsen pain itself, leading into a vicious cycle [5].

The goal of pain management is to attenuate pain to a level that allows for an acceptable quality of life (QoL) during all the phases of treatment [5,6,7,8,9,10]. Initial and ongoing assessment of pain should be a pivotal part of cancer care so as to guide treatment for pain relief. Pharmacological interventions are the mainstay of cancer pain therapy, often associated with other interventions, including at the physical level (e.g., surgery, radiation) as well as with other complementary strategies (e.g., psychological interventions). In 1986, the World Health Organization (WHO) developed a three-step “Analgesic Ladder”, which is still used as a teaching tool and a general guide to pain management. The analgesic ladder is based on a sequential approach from non-opioids, namely paracetamol and non-steroidal anti-inflammatory drugs (NSAIDs, step 1), to weak opioids (step 2) to strong opioids (step 3), according to pain intensity [5]. More recent guidelines propose a similar approach, with non-opioids used for mild pain, weak opioids or low dose of strong opioids for moderate pain, and strong opioids for severe pain [5,9,11]. On top of this, treatment of cancer pain comes with its own downfalls such as side effects of analgesic drugs, introducing new layers of complexity to cancer care. For this reason, cancer pain management should be encompassed by an integrated system for tailored, person-centered palliative care overseen by a multidisciplinary team [5,6,7,8,9,10].

Despite the availability of several guidelines on the matter [5,9,11,12,13], undertreatment of cancer pain is common worldwide. Around 80% of people dying from cancer experience moderate or severe pain lasting on average for 90 days when treatment coverage is low or absent [14]. A pan-European survey revealed that various types of pain or pain syndromes occurred across all stages of cancer, with inadequate treatment reported in a substantial proportion of patients, ranging between 56% and over 80% [15]. A systematic review reports that one-third of patients do not receive appropriate analgesia proportional to their pain intensity [8]. Cancer pain thus appears to be a major cause of unnecessary suffering.

Regarding Italy, there are areas that need improvement, such as adherence to guidelines, focus on the patient’s QoL, collaborative teamwork approaches, better assessment, and timely involvement of pain specialists, among others [16]. In light of these considerations, we conducted a national survey targeting Italian key healthcare professionals, including oncologists, radiotherapists, surgeons, and oncology nurses, to ensure a comprehensive multidisciplinary perspective. The aim of this study was to describe how oncology professionals assess and manage cancer pain in their daily clinical practice so as to identify educational needs when the reported clinical practice diverges from available guidelines and recommendations.

## 2. Methods

A descriptive research design was applied to this study. A multidisciplinary Scientific Board (SB) was formed in December 2023 to draft the survey, which consisted of a structured, self-administered questionnaire. The SB included nine healthcare professionals with direct expertise in oncology in oncological pain management, namely six medical oncologists (SC, PB, AA, LB, DS, FA), one surgeon (LM), one radiation oncologists (CF), and one oncology nurse (SG). The content validity of the survey was assessed by the SB to ensure its comprehensiveness, appropriateness, terminology, organization, and accuracy. The relevance and clarity of the survey questions were further refined through a pilot launch. Feedback and comments from this pilot phase led to minor revisions of the survey tool to enhance the clarity and comprehension of its items. Data collected during the pilot phase were excluded from the final analysis.

The survey question types were varied, including multiple-choice, ranking, and items allowing multiple responses to capture nuanced professional practices in pain management. The survey included a total of twenty-eight items divided in six categories, namely: (a) sociodemographic information and professional background, containing six items to assess basic demographic and professional data, including gender, age range, medical specialization, years of experience in oncology, and specific experience with pain therapy or palliative care (responses were captured via multiple-choice questions); (b) pain assessment tools, including two items evaluating familiarity and frequency of use of various pain assessment scales and questionnaires (responses were captured via multiple-choice answers and allowed multiple selections to reflect common practices in clinical settings); (c) perception of pain during disease phases, consisting of six items investigating perceptions regarding pain prevalence across different stages of disease such as diagnosis, active therapy, follow-up (it included both multiple-choice and conditional items, where respondents answered based on prior responses); (d) pharmacological pain treatment and its management, comprising seven items on pharmacological prescription practices (with a focus on opioids), including an ordinal ranking task to prioritize frequently used opioids and multiple-choice questions to assess considerations for opioid choice based on patient-specific factors; (e) adverse effects of opioid therapy, including five items addressing the adverse effects of opioid therapy and prophylactic treatments used in clinical settings (items were multiple-choice with options for selecting multiple applicable responses); and (f) challenges and solutions in pain therapy, comprising two items designed as multiple-choice questions allowing multiple responses, to identify barriers to effective pain management and proposed solutions for improving multidisciplinary collaboration.

With the exclusions of the items addressing the prescription of drugs, which were aimed at physicians, all other items were mandatory.

The survey was implemented online and was shared in March 2024 with the Italian Network of Supportive Care in Oncology (Network Italiano Cure Supporto in Oncologia, NICSO) to ensure maximum visibility among professionals working in the oncological setting and was open until May 2024.

After the closure of the survey, the anonymous individual responders’ data were pooled and analyzed with descriptive statistics. Responses to multiple-choice were normalized to the total number of responses. To evaluate the relationship between two or more categorical variables, contingency tables were used to organize the data, followed by statistical tests such as the chi-square test, which assessed the strength of the associations between the variables. The ranking preferences of clinicians for different analgesics (Oxycodone, Fentanyl, Morphine, Tapentadol, Buprenorphine, Hydromorphone, and Methadone) were analyzed using the Kruskal–Wallis test, followed by Dunn’s post-hoc analysis to identify statistically significant differences between the various opioid treatments. *p*-values < 0.05 were considered statistically significant.

## 3. Results and Discussion

### 3.1. Group Study Features

Eighty-five NICSO members responded to the survey; their demographic and job characteristics are reported in Table 1. The data show that the study group is relatively young, with the majority of participants aged between 30 and 45 years. The participants’ professional profile is predominantly composed of oncologists (*n* = 54, 63.5%), but there is also a good representation of nurses (*n* = 15, 17.7%) and radiation oncologists (*n* = 12, 14.1%), while surgeons are underrepresented (*n* = 4, 4.7%). Additionally, 20 participants (23.5%) (especially medical oncologists) reported having attended master’s or specialized courses in pain therapy or palliative care. The analysis revealed a strong relationship between the clinician’s years of experience and their attendance at master’s or specialized courses (Chi^2^ = 123.4, *p* < 0.0001). It has been observed that participants with more than 10 years of experience reported the highest rate of attendance (95%) compared to those with fewer years of experience (20%). This could suggest that further education becomes more critical as participants face more complex clinical situations or gain a deeper understanding of the nuances of pain management. Fifty-eight (68.2%) of the respondents work in an outpatient setting, whereas the remaining operate in an inpatient setting (Table 1).

The SB’s discussion revealed that the low representation of surgeons is justified by the fact that these professionals are rarely involved in the supportive and palliative care setting of patient management and predominantly focus on other clinical outcomes such as overall survival and disease-free survival rates, rather than on the pain that can affect the patient’s QoL.

Regarding the work setting, the underrepresentation of the inpatient setting could pose a limitation of the study, as inpatient care offers more opportunities to observe the progression of pain following analgesic treatment, compared to the outpatient setting. However, it is important to consider that not all oncology departments include inpatient care, and the low representation might be because many facilities do not provide this option to patients.

### 3.2. Pain Assessment Tools Routinely Used in Clinical Practice

The assessment of pain from diagnosis throughout disease course must be integrated into routine cancer care. Data on the use of pain assessment tools (85 respondents, 102 total answers) indicate that pain during the course of the disease is assessed by the majority of professionals using evaluation scales (*n* = 84, 82.4%), either alone or in combination with other assessment tools, such as specific scales for breakthrough cancer pain (BCP) (*n* = 9, 8.8%), the 4-item Neuropathic Pain Questionnaire (DN4) [17] (*n* = 5, 4.9%), and other “specific instruments” (*n* = 4, 3.92%). The data indicate a significant relationship between years of clinical experience and the number of evaluation scales used (Chi^2^ = 29.94, *p* < 0.0001). Participants with intermediate experience (between 5 and 20 years) tend to use 2 or more scales more frequently. Among the evaluation scales (84 respondents, 126 total answers), the Numerical Rating Scale (NRS) is the most commonly used (*n* = 75, 60.3%), followed by the Visual Analog Scale (VAS) (*n* = 41, 32.5%) and the Verbal Rating Scale (VRS) (*n* = 9, 7.1%), in line with literature data [18]. Indeed, the European Society for Medical Oncology (ESMO) guidelines recommend screening the intensity of pain regularly and consistently using the VAS or NRS using the question: ‘What has been your worst pain in the last 24 h?’ to indicate if additional, comprehensive assessment is needed (weak recommendation) [9].

The analysis revealed that the use of specific questionnaires for BCP is associated with years of experience, with participants who have more experience (>10 years) being more likely to use them at least once (Chi^2^ = 80.66, *p* < 0.0001). However, overall usage is low across all experience groups. Among respondents who reported using specific scales for BCP, the Breakthrough Pain Assessment Tool (BAT) (*n* = 6, 50%) [19] and the Breakthrough Pain Questionnaire (*n* = 4, 33.3%) were the most utilized, followed by the Italian Questionnaire for Intense Episodic Pain (QUDEI) (*n* = 2, 16.7%) [20], either alone or in combination. Among the four respondents who selected “specific tools”, all reported using the Edmonton Symptom Assessment Scale (ESAS).

These findings suggest that specific questionnaires are challenging to administer in outpatient settings due to the limited time healthcare providers have for each patient visit. This may explain the preference for using assessment scales, which are easier to administer.

However, in outpatient settings, these scales are not typically included by default in the patient’s medical records. This may be due to limited awareness about the questionnaires, of which there are several and are not all easily applicable in outpatient contexts. The implementation of some assessment scales in a web/app format is proposed, which would facilitate score calculation of the scale for healthcare providers upon completion. In contrast, the diverse use of assessment tools is more common in inpatient settings, where patients are continuously monitored, allowing for pain variations to be measured over time. An important consideration is that the availability of multiple pain assessment questionnaires may complicate training, as teaching HCPs to use a wide array of tools effectively is more challenging than focusing on a select few. This increased complexity may lead to inconsistencies in pain assessment and, consequently, a higher likelihood of suboptimal pain management. All professionals should thus initially receive basic training on evaluation scales, followed by more specialized training on specific scales at a later stage.

In this perspective, the ESAS could be a valid candidate due to its shortness and ease of use [21,22]. A robust retrospective matched cohort study using ESAS at different time points (initial, treatment and palliative, advanced phases) and enrolling almost 130,000 patients showed higher 5-year survival rates for assessed patients compared to non-assessed ones, and ESAS was significantly associated with a decreased mortality risk, suggesting that regular symptom evaluation enables early identification and management of symptoms and treatment-related side effects. This proactive approach likely mitigates symptom burden, prevents complications, and optimizes overall patient care, thus contributing positively to survival outcomes. This study provides compelling real-world evidence of the value of routine symptom assessment in enhancing cancer care throughout the disease trajectory [23]. The ESAS has been validated for the Italian language [24] and subsequently modified to develop the ESAS-Total Care (ESAS-TC) by adding three questions to the original version: financial disease-related preoccupations; spiritual pain (deep, interior suffering); and social isolation [25]. The ESAS-TC will potentially help move cancer research toward personalized total cancer care at any stage of the disease.

### 3.3. Pain Intensity in the Different Stages of the Disease

Nearly all survey participants (*n* = 82, 96.5%) reported that the percentage of patients experiencing any level of pain or moderate-to-severe pain (defined as VAS > 4/10) varies across different stages of the disease (i.e., diagnosis, active therapy, follow-up, or recurrence), with a very small minority (*n* = 3, 3.5%) reporting that pain intensity remains constant.

**Figure 1:**
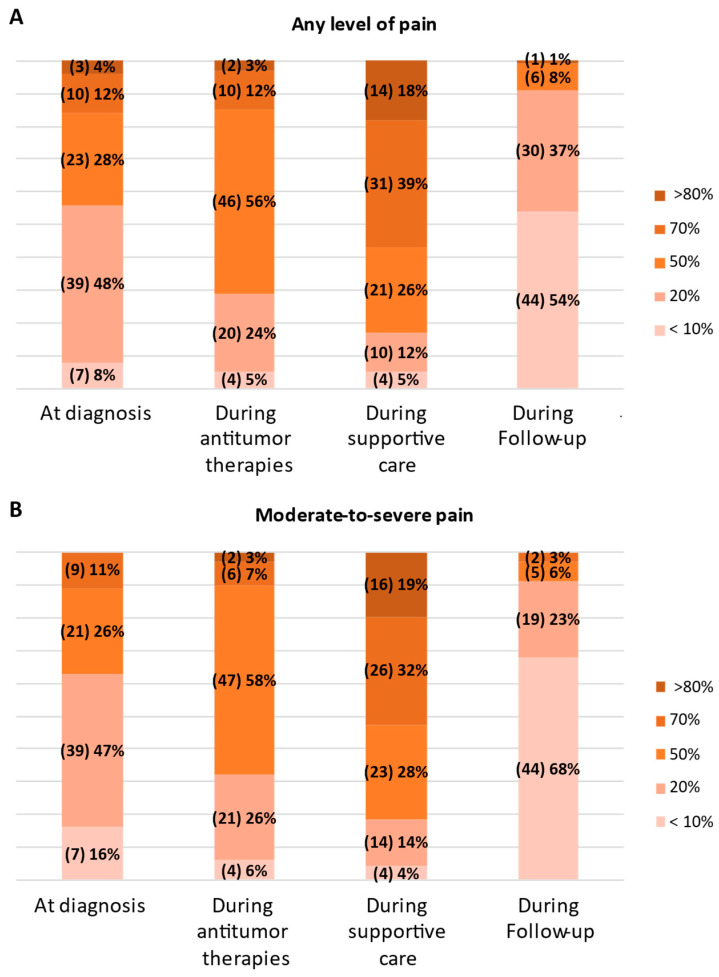
Any (**A**) and moderate-to-severe (**B**) pain across disease trajectory according to the survey respondents.

Based on the participants’ reports, the estimated overall pain prevalence across various stages of the disease was 39%, with a standard error (SE) of 0.3%. Interestingly, over 50% of respondents report that only up to 20% of patients experience any level of pain or moderate-to-severe pain at diagnosis (Figure 1A,B). The percentage of patients experiencing any level of pain or moderate-to-severe pain increases to 50–70% during antitumor therapies, estimated by 68% and 65% of participants, respectively (Figure 1A,B). This could be explained by including patients with recurrent disease undergoing multiple therapy cycles. During supportive care, most participants believe that 50–80% of patients experience any pain or moderate-to-severe pain. Finally, during follow-up, almost the totality of participants indicated that the percentage of patients with any pain or moderate-to-severe pain drops below 20% (Figure 1A,B). These findings may partially contrast with existing literature, particularly at the diagnostic and follow-up stages. However, the available literature is inconsistent and highly heterogeneous regarding the assessment of pain experienced by oncological patients at different stages of disease. A recent meta-analysis reported a slightly higher prevalence of moderate-to-severe pain compared to our findings, ranging from 28% to 42% at the diagnostic stage [26], indicating a potential underestimation of patient-perceived pain in our findings. Conversely, the meta-analysis showed similar pain prevalence during treatment and supportive care [26]. This observation is partially aligned with a previous systematic review, which found that 59% of patients experienced pain during cancer treatment, and 64% in metastatic, advanced, or terminal disease [2]. After curative treatment, the prevalence of any type of pain in oncological patients ranges from 9% to 87% [2,26]. In 2022, another meta-analysis reported an overall pain prevalence of 40% during and up to three months after curative cancer treatment, with a heterogeneity of 96% [27]. Several factors, including treatment modalities [28], timing of assessment [29], and measurement tools [28,30], could influence pain prevalence rates. These discrepancies might be addressed by better defining how survey respondents interpret follow-up stages (e.g., radically treated disease, absence of disease, etc.).

In addition, post-treatment or post-surgical iatrogenic pain might be underestimated by some professionals, who tend to focus on treatment success rather than post-intervention patients’ QoL. To address this gap, it is essential to assess pain consistently at the beginning and conclusion of each treatment phase [27]. This requires clear cut-offs for mild, moderate, and severe pain levels [31], as well as an evaluation of the worst pain experienced by the patient in the past 24 h [9]. Furthermore, comprehensive pain assessment should incorporate psychosocial factors that influence the patient’s perception and experience of pain [32]. These factors, alongside the assessment of pain, can provide a more holistic understanding of the patient’s condition and improve the overall management of pain.

The discrepancies between literature data and the survey’s results may be explained by the fact that HCPs, as reported above, often do not use specific pain assessment questionnaires due to lack of time, insufficient knowledge, or because they do not consider them useful; moreover, they may be also influenced by the type of cancer and interventions, which can significantly affect pain perception; e.g., it has been showed that patients with prostate and bladder cancer reported less pain (26–27%) than patients with head and neck cancer (48%) or patients with cancer of the female genital organs (45%) [32]. Bridging this gap could involve the routine introduction of pain assessment tools to better capture pain levels, also allowing data pooling on large cohorts. For these reasons, a transdisciplinary educational approach is recommended, focusing on training new generations of professionals across all specialties for improved clinical practice. The inclusion of pain assessments of iatrogenic pain in follow-up visits should also be encouraged. Future studies should further analyse patients based on cancer type and administered therapies as well.

### 3.4. Therapeutic Options Used in Clinical Practice

The therapeutic options used in clinical practice are reported in Table 2 (71 respondents for a total of 218 answers). In general, when considering factors for selecting the most appropriate medications (Table 2, question A), participants most frequently highlighted patients’ comorbidities (66 answers, 30.1%) and the use of multiple medications (61 answers, 28.0%). This is in line with European guidelines recommending specific formulations depending on the patient’s comorbidity, such as the use of transdermic or intravenous fentanyl and buprenorphine in patients with chronic kidney disease [9]. Patients’ age and the presence of a caregiver were also important. Additionally, a good portion of respondents (*n* = 28, 38.43%) indicated that all listed factors should be considered. These findings are consistent with another Italian survey, which reported that analgesic treatment is typically initiated based on a comprehensive clinical evaluation, taking into account the characteristics of the pain and patients’ comorbidities and ongoing treatments [16].

The key aspects of opioid therapy, as perceived by professionals, are summarized in Table 2 (question B). The preferred route of administration is oral (38 answers, 17.5%), but only a minority of respondents recommend avoiding the parenteral route (10 answers, 4.6%). Administering medications at fixed interval accounted for 47 answers (21.7%). Additionally, in about a tenth of the cases, immediate-release titration to achieve the minimum effective dose, the introduction of adjuvant medications such as corticosteroids, and the use of paracetamol were deemed necessary. The most voted item (61 answers, 28.11%) was related to the use of a rescue dose of 15–20% of the single dose or 1/6 of the total daily dose when pain is not adequately controlled throughout the day. ESMO guidelines advocate for the prevention of pain onset by means of around-the-clock, oral administration, taking into account the half-life, bioavailability, and duration of action of different drugs, and suggest prescribing analgesics on a regular basis and not on an ‘as required’ schedule; when oral administration is not feasible due to conditions such as severe vomiting, bowel obstruction, severe dysphagia, or severe confusion, and in cases where pain control is inadequate requiring rapid dose escalation, or when oral opioids cause significant adverse effects, alternative routes of administration should be considered [9].

When initiating opioid treatment for severe pain (Table 2, question C), 28 (40.0%) respondents reported prescribing one of the commercially available drugs, calculating the dose based on their professional experience. The same percentage stated that they assess whether the patient is using weak opioids and begin titration with oral morphine. Eight (11.4%) continue using non-steroidal anti-inflammatory drugs (NSAIDs), both scheduled and as needed, while initiating low-dose opioid therapy. Finally, six respondents (8.6%) reported that they prescribe one of the available drugs and determine the dose based on their clinical experience, while avoiding as-needed therapies to prevent patient confusion; instead, they opt for slightly higher doses than those typically considered effective for that type of pain. In another Italian survey, opioid titration was reported as the preferred strategy to establish the most suitable drug regimen [16].

**Figure 2:**
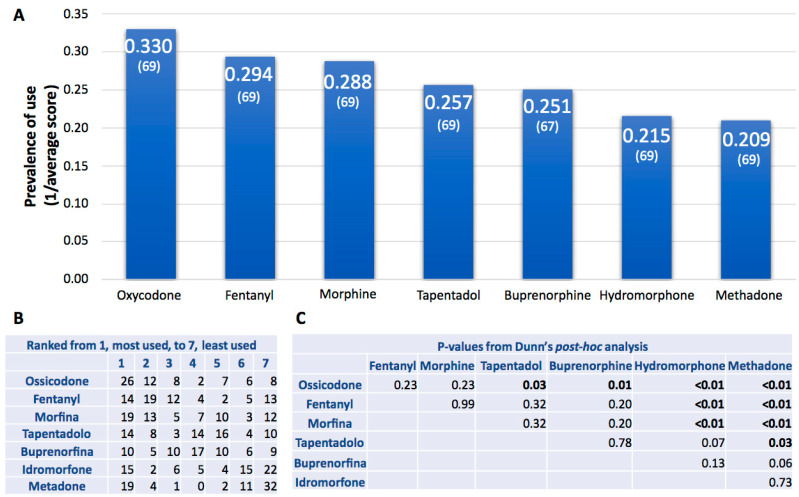
Prevalence of use of strong opioids in daily clinical practice. (**A**) Each drug was assigned a score from 1 (frequently used) to 7 (rarely used). The average score for each drug was calculated, and the inverse value was displayed as a histogram. The number of responses is shown in parentheses. (**B**) The table displays the number of respondents who assigned a score from 1 to 7 for each drug. (**C**) The statistical significance of the prevalence comparison between different treatments was determined using Dunn’s post-hoc analysis; the numbers in bold are statistically significant (*p* < 0.05).

When physicians were asked to rank opioids based on their frequency of use in daily clinical practice, from 1 (most frequently used) to 7 (least frequently used) (Figure 2A,B), the analysis revealed a significant difference in rankings among the opioids (*H* = 32.35, *p* = 0.00001). Oxycodone emerged as the most frequently used opioid, showing significant differences compared to tapentadol (*p* = 0.03), buprenorphine (*p* = 0.01), hydromorphone, and methadone (*p* < 0.01) (Figure 2C). Fentanyl and morphine followed oxycodone in the frequency of use, with both demonstrating significant differences compared to hydromorphone and methadone (*p* < 0.01, Figure 2C). Tapentadol and buprenorphine were also commonly used, primarily ranked between 3 and 5. Among these, only tapentadol exhibited a significant difference in prevalence of use compared to methadone (*p* = 0.03). Methadone and hydromorphone emerged as the least used opioids, with most rankings being 6 and 7 (Figure 2A–C). These results are partially in contrast with major international guidelines, which recommend oral morphine as the opioid of first choice [5,9]. However, the available literature also demonstrates variability. For instance, one study reported that morphine and tramadol were the most used opioids in cancer pain management [33], while another survey indicated that all participating centers managed mild to moderate pain with paracetamol, NSAIDs, and tramadol, and severe pain using strong opioids (i.e., buprenorphine and fentanyl) [34].

The observation that some survey respondents are inclined to both prescribe one of the available medications and determine the dose, while also avoiding as-needed therapies to prevent patient confusion by prescribing slightly higher doses than those considered effective for that type of pain, highlights a strong empiricism in the treatment of pain with opioids. This underscores the need to strengthen the foundational knowledge of this subject and to follow precise and structured guidelines. Nonetheless, dynamic, multidimensional and personalized approaches might be better suitable to tailor the therapy to the patient’s needs, especially for opioid-naïve patients [35,36].

#### 3.4.1. Rescue Dose for Patients on Existing Opioid Therapy with Inadequately Controlled Pain

According to 49 (74.2%) respondents, the rescue dose for patients already on opioid treatment with inadequately controlled pain is approximately 1/6 of the total daily opioid dose, similar to what is indicated by the American Society of Clinical Oncology (ASCO) and ESMO guidelines [9,11]. Eight (12.1%) indicated that there is no recommended dosage, five (7.6%) reported using half of the total daily opioid dose, and three (4.5%) between 50% and 60% of the fixed dose, as indicated by the WHO [5]. Only one reported using a rescue dose of 10 mg of subcutaneous morphine (Table 3, question D). Immediate-release formulations, characterized by a quick onset of analgesia and brief duration, are commonly recommended for use as rescue medications. If multiple rescue doses are required daily, it is important to reassess and adjust the baseline opioid therapy accordingly [9].

#### 3.4.2. Perception of Scientific Evidence on the Use of Cannabis for Severe Pain

Data on the perception on the use of cannabis for severe pain are reported in Table 2 (question E). Several survey participants (*n* = 27, 39.1%) believe that cannabis use for the treatment of severe pain is supported by sufficient scientific evidence; conversely, 19 (27.5%) do not think there are enough data in the literature. A smaller proportion (*n* = 9, 13.04%) suggest that the scientific evidence depends on the type of cannabinoid considered (e.g., THC, CBD, CBN, etc.). Additionally, 5 (7.3%) believe that the evidence demonstrates cannabis efficacy only for neuropathic pain, while 3 (4.4%) see it as effective only in recurrent/metastatic disease (Table 2, question E). Among the six participants who selected “Other”, four stated that they do not know, and two indicated that cannabis’s analgesic indication is mainly for non-oncological conditions, such as spasticity in multiple sclerosis or for alleviating symptoms like nausea and anorexia [9].

This perception that the available data on cannabis are sufficient to support its use in pain treatment contrasts with the Multinational Association of Supportive Care in Cancer (MASCC) and ESMO guidelines [9,37], which indicate that current scientific evidence is not robust and suggest further trials to clarify the role of cannabis when conventional therapy fails.

It is crucial to raise awareness among professionals about this discrepancy and the need for higher-quality clinical studies. SB members’ past experiences reveal that patients rarely request therapeutic cannabis. Instead, some clinicians propose cannabis, particularly for neuropathic pain, highlighting a cultural divide that complicates standardizing clinical studies. Therefore, enhanced professional training and well-structured studies involving homogeneous patient populations and standardized therapies are essential. This issue was also highlighted in a recent Belgian survey, which reported that while an active teaching program on pain relief is offered in 66% of the participating centers, only 33% of these centers actively conduct research focused on pain management [34].

### 3.5. Opioid Treatment: Adverse Events and Their Prevention

Among the participants, almost the totality (*n* = 82, 96.5%) agreed that constipation is the primary side effect of opioid treatment, followed by drowsiness, confusion, hallucinations, and myoclonus (*n* = 61, 71.8%), nausea and vomiting (*n* = 59, 69.4%), respiratory depression (*n* = 4, 4.7%), and finally pruritus (*n* = 3, 3.5%). According to the SB, pruritus is an underreported side effect compared to others.

The administration of prophylactic treatments concomitantly with opioids to alleviate the most common side effects is recommended in current clinical settings [33]. In line with this observation, most participants (*n* = 74, 87.1%) reported using preventive therapies to counteract such adverse events. Commonly used preventive measures (74 respondents, 131 answers) include laxatives (69 answers, 52.7%) and antiemetics or H2 receptor agonists (53 answers, 40.5%). Mood stabilizers, neuroleptics, and benzodiazepines are prescribed less frequently (four, three, and two answers, respectively). These preventive measures are administered both as monotherapy and in combination.

Since opioid-induced constipation (OIC) affects 40% to 90% of patients treated with opioids [38,39], the routine prescription of laxatives against OIC is strongly recommended [9,38,39]. Regarding the use of laxatives for OIC prophylaxis (68 respondents, 122 answers), the most commonly used are osmotic agents (54 answers, 44.3%), followed by bulk-forming agents (23 answers, 18.9%), stimulant agents (21 answers, 17.2%), and microenemas/suppositories (21 answers, 17.2%); saline agents are hardly prescribed (2 answers, 2.5%). It was also reported that different types of laxatives are prescribed both as monotherapy and in combination. The survey participants (67 respondents) reported managing OIC mostly with naldemedine (*n* = 37, 55.2%) and naloxegol (*n* = 22, 32.8%), with enemas, no medication, or methylnaltrexone bromide reported only in a minority of cases (3, 2, and 1 answers, respectively). Of note, guidelines suggest the use of osmotic or stimulant laxatives and do not recommend bulk ones; in case of unresolved OIC, peripheral opioid antagonists (i.e., methylnaltrexone or naloxegol) can be of use [38]. The literature does not indicate the superiority of one laxative over another [38]; therefore, the choice is left to the discretion of the prescribing professional. In this context, the survey responses and the encouraging prescription rates of laxatives and antiemetics or H2 receptor antagonists reflect good clinical practices.

### 3.6. Causes of Unsatisfactory Outcomes in Cancer Pain Therapy and Possible Solutions

The potential causes for unsatisfactory outcomes in multidisciplinary oncologic pain management and possible solutions, as highlighted by responders, are reported in Table 3. The main factors negatively impacting cancer pain management include inadequate training (59 answers, 85.5%) as well as poor communication between healthcare professionals and patient caregivers (46 answers, 66.7%), between patients and families/caregivers (28 answers, 40.6%), and within the care team itself (22 answers, 31.9%). Poor communication between healthcare professionals and patient caregivers can result in caregivers being unprepared to manage pain effectively, leading to improper medication administration or delays in seeking help for symptom escalation. Enhancing communication in this area should focus on educating caregivers about the prescribed pain management plan, including medication schedules, side effect monitoring, and when to contact healthcare providers. Similarly, communication gaps between patients and their families or caregivers may stem from differing expectations or misunderstandings about the patient’s needs and treatment goals. Addressing this requires fostering an open dialogue that ensures caregivers and families are aligned with the patient’s preferences and informed about their role in supporting pain management. Finally, inadequate communication within the care team itself can lead to inconsistencies in treatment approaches, delays in decision-making, and fragmented care. Improving this requires establishing clear protocols and regular interdisciplinary meetings to ensure all team members, including doctors, nurses, and allied health professionals, are aligned in their approach to pain management. It is thus essential to provide education that involves not only medical staff but also patients and caregivers, regardless of their professional background. In this context, patient organizations could play a crucial role in facilitating communication by acting as mediators to bridge the gap between healthcare providers and patients/caregivers. They can provide resources, organize workshops, and support groups to enhance caregivers’ understanding of cancer pain management while empowering patients to voice their needs and concerns effectively.

Scientific societies should be engaged by encouraging their members to continually improve their skills through training courses and the development of supportive educational tools (e.g., informational brochures) and support adherence to guidelines [16].

It is also important to define a structured approach to training that includes both theoretical knowledge and practical field experience, possibly certified. Interprofessional internships could be promoted to foster communication between students and healthcare professionals, preparing learners from the outset for a collaborative approach that overcomes the barriers often present within the care team. Therefore, the importance of adopting a multidisciplinary approach to improve cancer pain management is emphasized.

### 3.7. Call to Actions

- Raise awareness that pain is a critical issue to be addressed from the time of diagnosis and is not confined to the advanced stages of the disease.
- Pain assessment should become a routine task starting at diagnosis, regardless of the clinical setting—whether inpatient or outpatient.
- Promote a proactive approach to pain assessment (and subsequent management), as this reduces symptom burden, prevents complications, and ultimately enhances the overall quality of care.
- Support the use of simple and user-friendly questionnaires (such as the ESAS) to assess pain consistently to better define pain levels across different cancer types and disease stages and allow data pooling to obtain solid real-world data.
- Support the implementation of such questionnaires in a web/app format to streamline pain assessment, especially in the outpatient setting.
- Adopt a multi-level, multidisciplinary approach involving all stakeholders: educators to foster interprofessional training and continuous education programs, patient organizations, and scientific societies.
- Guarantee foundational training in pain assessment and management, as well as specialized training, following the model of generalist versus specialist palliative care, where all professionals are expected to have basic competencies. Training should include theoretical knowledge and practical field experience, as well as interprofessional internship (possibly certified).
- Better engage scientific societies and universities by encouraging members to continually improve their skills through development of supportive educational tools and additional training courses.
- Improve communication and education within HCPs, patients, and caregivers, especially regarding the correct use of opioids; providing information on opioid safety to patients and caregivers could help promote adherence to therapy.
- Address the empiricism often seen in pain management by encouraging multicenter and, most importantly, interdisciplinary research initiatives.
- Raise awareness on the actual use of therapeutic cannabis and support the implementation of well-structured studies with homogeneous patient populations.

## 4. Conclusions

This survey offers valuable insights into the current practices and challenges in cancer pain assessment and management among Italian oncology professionals. While adherence to pain management guidelines and the adoption of multimodal strategies were evident in several areas, key gaps remain, particularly in the consistent use of validated pain assessment tools and the integration of multidisciplinary approaches. To our knowledge, this is one of the very few survey-based studies addressing these clinical needs, providing a unique perspective on healthcare professionals’ viewpoints. It underscores the persistent underestimation of pain assessment and perception in oncological patients, highlighting the urgent need for enhanced focus on accurately evaluating and addressing patients’ pain experiences across all stages of their care journey. However, some limitations should be considered when interpreting these findings. The survey predominantly involved a younger, outpatient-based group of oncology professionals, with a strong representation of oncologists and limited involvement from surgeons and inpatient settings. This demographic and professional skew may limit the generalizability of the results to broader clinical contexts. Moreover, while the survey was distributed through NICSO—whose extensive network likely facilitated diverse participation—this distribution method may have introduced biases tied to practices specific to NICSO-affiliated institutions. Additionally, the online nature of the survey inherently carries potential biases that warrant attention. For instance, recall bias could arise as participants might inaccurately recall past experiences or generalize their practices, especially in retrospective questions. Selection bias is another concern, as online surveys may attract participants who are more engaged and technologically adept, resulting in non-representative sampling. Response bias may also pose a risk, as participants might rush through the survey or misinterpret questions due to format, language, or complexity, leading to random or invalid responses. To strengthen the reliability and applicability of these findings, future studies should incorporate complementary methodologies, such as in-depth interviews, focus groups, or observational studies, to validate the survey data and provide richer contextual insights.

Despite these limitations, the current findings remain significant and underscore an urgent need to enhance education and training to equip healthcare providers with the skills necessary to assess and manage cancer pain effectively and expand research initiatives alongside better communication and collaboration within care teams and with patients/caregivers.

Future efforts should prioritize cultivating a robust multidisciplinary culture, implementing structured training programs, and encouraging the active involvement of scientific societies in the development of educational resources and evidence-based strategies. By addressing these challenges, healthcare systems can significantly improve the quality of care for cancer patients, reducing the burden of pain and enhancing patients’ overall quality of life throughout their disease trajectory.

## Figures and Tables

**Table 1:** Personal and professional characteristics of the survey respondents.

Questions	Available Answers	Answers, *n* (%)
Sex	Male	31 (36.5)
	Female	53 (62.4)
	I’d rather not disclose	1 (1.2)
Age	<30 years	21 (24.7)
	30–45 years	47 (55.3)
	45–60 years	14 (16.5)
	>60 years	3 (3.5)
Years of experience in the oncological setting	<5 years	39 (45.9)
	10–20 years	17 (20.0)
	5–10 years	15 (17.7)
	>20 years	14 (16.5)
Professional profile	Medical oncologist	54 (63.5)
	Nurse	15 (17.7)
	Radiation oncologists	12 (14.1)
	Surgeon	4 (4.7)
Respondents who attended a master’s or specialization courses in pain therapy or palliative care	Yes	20 (23.5) ^a^
	No	65 (76.5) ^b^
Work setting	Inpatient care	27 (31.8)
	Outpatient care	58 (68.2)

^a^ Medical oncologist, *n* = 13; Nurse, *n* = 6; Surgeon, *n* = 1. ^b^ Medical oncologist, *n* = 41; Nurse, *n* = 9. Radiation oncologists, *n* = 12; Surgeon, *n* = 3.

**Table 2:** Therapeutic options used in cancer pain clinical practice according to the survey participants.

	*n* (%)
A. In your clinical practice, when initiating opioid therapy, what additional factors, beyond the specific type of pain (e.g., neuropathic, nociceptive, visceral, mixed, etc.), do you consider in selecting the appropriate medication? (multiple answers allowed; 71 respondents, 218 total answers)	
1. Patient comorbidities (e.g., diabetes, cardiovascular disease, chronic kidney disease, etc.)	66 (30.3)
2. Polypharmacy and interactions with other medications the patient is already taking	61 (28.0)
3. Age	47 (21.6)
4. Presence of a caregiver	44 (20.2)
5. No other factors are considered	0 (0)
B. What do you consider to be the essential aspects of opioid therapy? (multiple answers allowed; 68 respondents, 217 total answers)	
1. Consider a rescue dose (+15–20% of the single dose or 1/6 of the total daily dose) when pain is not adequately controlled throughout the day.	61 (28.1)
2. Administer medications at fixed intervals.	47 (21.7)
3. Prefer oral administration whenever possible.	38 (17.5)
4. Perform titration with immediate-release formulations to determine the minimum effective dose.	23 (10.6)
5. Always introduce adjuvant medications (e.g., corticosteroids).	20 (9.2)
6. Continue the use of paracetamol.	18 (8.3)
7. Avoid parenteral administration routes.	10 (4.6)
C. When initiating opioid treatment for severe pain, what strategy do you employ? (70 respondents)	
1. I prescribe one of the commercially available medications and calculate the necessary dose based on my personal experience.	28 (40.0)
2. I assess whether the patient has been taking weak opioids and begin titration with oral morphine.	28 (40.0)
3. I continue using NSAIDs on a scheduled basis and as needed and start the patient on low-dose opioids.	8 (11.4)
4. I avoid prescribing as-needed therapies to prevent patient confusion and prescribe doses slightly higher than what I consider effective for that type of pain.	0
5. Answers 1 and 4	6 (8.6)
D. In your clinical practice, approximately what rescue dose is used for patients already on opioid treatment with inadequately controlled pain? (66 respondents)	
1. Approximately 1/6 of the total daily opioid dose	49 (74.2)
2. No recommended dosage	8 (12.1)
3. Half of the total ongoing daily opioid dose	5 (7.6)
4. Between 50% and 60% of the regular dose	3 (4.6)
5. 10 mg of subcutaneous morphine	1 (1.5)
E. Do you believe that the use of cannabis in severe pain is supported by sufficient scientific evidence? (68 respondents)	
Yes	27 (39.1)
No	19 (27.5)
It depends on the type of cannabinoid	9 (13.0)
Other	6 (8.7)
Only for neuropathic pain	5 (7.3)
Only for patients with recurrent/metastatic disease	3 (4.4)
NSAIDs, nonsteroidal anti-inflammatory drugs	

All questions were provided with predefined answer options, and in certain cases, respondents could input subjective responses using the ’Other’ option (i.e., question E). Questions A and B allowed multiple responses, and the data were normalized to the total number of responses. Questions C to E allowed only one response, and the data were normalized to the total number of respondents.

**Table 3:** Reasons and solutions of unsatisfactory outcomes in oncologic pain therapy according to the survey participants.

Answers	*n* (%)
A. According to you, what are the possible causes of unsatisfactory outcomes in oncologic pain therapy?	
Inadequate training among healthcare providers	59 (85.5%)
Poor communication between healthcare providers and patients/caregivers	46 (66.7%)
Poor communication between patients and their families/caregivers	28 (40.6%)
Poor communication within the care team/doctor-nurse communication	22 (31.9%)
Individual environmental barriers	14 (20.3%)
Lack of access to appropriate medications	11 (15.9%)
Individual genetic barriers	5 (7.3%)
Other factors (e.g., patient and family reluctance to use opioids, participation in palliative care programs, anxiety about specific medications, patient non-adherence to treatment, concerns about morphine derivatives)	3 (4.4%)
B. Please indicate the solutions you consider most effective for improving the multidisciplinary pain management in oncologic patients.	
Establishing a dedicated hospital pathway for managing oncologic pain	63 (22.9%)
Continuous collaboration with general practitioners in pain management	61 (22.2%)
Discussing complex cases with pain management specialists	61 (22.2%)
Involving nurses in symptom screening, evaluating therapeutic effectiveness, and monitoring potential side effects	53 (19.3%)
Engaging pain management specialists when patients start experiencing pain	36 (13.1%)
Other (referring patients to simultaneous palliative care clinics from the outset, regardless of pain presence)	1 (0.4%)

All questions were provided with predefined answer options, and in certain cases, respondents could input subjective responses using the ’Other’ option (i.e., question B). Question A allowed multiple responses, and the data were normalized to the total number of respondents (*n* = 85). Question B allowed multiple responses, and the data were normalized to the total number of responses (*n* = 275).

## Data Availability

Data will be available upon reasonable request to the corresponding author.
